# Impact of Telemedicine on Health Expenditures During the COVID-19 Pandemic in Japan: Quasi-Experimental Study

**DOI:** 10.2196/72051

**Published:** 2025-09-23

**Authors:** Hikaru Aihara, Ichiro Kawachi, Benjamin D Sommers

**Affiliations:** 1 Department of Health Policy and Management, Harvard T.H. Chan School of Public Health, Boston, MA, United States, 1 617 495 1000; 2 Department of Public Health and Health Policy, Graduate School of Medicine, The University of Tokyo, Bunkyo Hongo 7-3-1, Tokyo, 113-0033, Japan, 81 35841 3491; 3 Department of Social and Behavioral Sciences, Harvard T.H. Chan School of Public Health, Boston, MA, United States; 4 Department of Medicine, Brigham and Women’s Hospital, Boston, MA, United States

**Keywords:** telemedicine, health reform, health spending, cost containment, COVID-19

## Abstract

**Background:**

The effects of telemedicine on health expenditures and health outcomes are an important policy question. Many countries loosened regulations on the use of telemedicine during the COVID-19 pandemic, thereby offering an opportunity to evaluate these effects via a natural experiment.

**Objective:**

This study aimed to assess the effect of greater telemedicine use on area-level health expenditures and health outcomes related to common chronic conditions in Japan during the COVID-19 pandemic.

**Methods:**

We compared prefectures (area levels of government) with higher prepandemic telemedicine rates (fiscal year [FY] 2019) versus those with lower rates and conducted a difference-in-differences analysis of the change in prefecture-level health expenditures from FY2017 to FY2022 and health outcomes from FY2017 to FY2021. The participants were the total population in Japan from FY2017 to FY2022 (n=126 million), and the exposure was the increase in telemedicine use following the government’s relaxation of restrictions on telemedicine use as an exceptional measure during the COVID-19 pandemic. Our main outcomes were the share of outpatient claims that were for telehealth services; total, inpatient, and outpatient annual prefecture-level health expenditures; all-cause mortality, glycated hemoglobin, systolic blood pressure, and low-density lipoprotein cholesterol.

**Results:**

Treatment prefectures (n=15, population of 62 million) were defined as those with greater-than-median telemedicine use before the pandemic, while control prefectures (n=32, population of 64 million) were defined as those with less-than-median telemedicine use. Treatment and control prefectures shared similar demographic characteristics before the pandemic. The growth in telemedicine after 2020 as a share of outpatient claims increased among the treatment prefectures by 0.35 percentage points more than among control prefectures, which represented more than a threefold increase in telemedicine use compared to the prepandemic median. In difference-in-differences analyses, this difference was associated with a 1.0% relative decrease (95% CI 0.3%-1.8%) in total health expenditure (*P*=.006) and a 1.1% relative decrease (95% CI 0.2%-2.0%) in inpatient expenditure (*P*=.02). Outpatient expenditures showed no significant difference as a result of increased telemedicine adoption. Most health outcomes—all-cause mortality, glycated hemoglobin, systolic blood pressure, diastolic blood pressure, and low-density lipoprotein cholesterol—did not show any significant changes.

**Conclusions:**

Areas in Japan with greater expansion of telemedicine use during the pandemic experienced a significant decrease in both inpatient and total health care spending compared with areas with less telemedicine use, without harming health outcomes.

## Introduction

A potential benefit of telemedicine is that it could save time and costs for patients and clinicians [1]. However, there have been few formal evaluations of the effects of telemedicine on total health spending despite high levels of interest from policymakers in many countries. A systematic review of the literature published between January 2020 and May 2022 concluded that 25 out of 31 original studies demonstrated cost reductions resulting from the use of telemedicine services, but nearly all of these studies focused on costs for patients and providers rather than the health care system as a whole [1]. For patients, telemedicine reduces travel time and the need to take time off work [2-6]. For health care providers, it reduces unscheduled visits and personnel costs, although there are initial set-up costs associated with telemedicine technology [7-14]. Medical costs were reported to decrease for various groups of specific patients, including patients with diabetes, patients with pacemakers, patients on peritoneal dialysis, patients with automated peritoneal dialysis, patients undergoing hip and knee arthroplasty, patients with anxiety disorders, children with medical complexity, and Medicare beneficiaries with major depression [15-21]; research on telemedicine for patients hospitalized for ischemic stroke showed a slight increase in health expenditures.

At the same time, attention must be paid to whether the treatment provided by telemedicine has negative impacts on patient health outcomes. Studies also found improvements in quality of care and user safety, such as positive safety outcomes compared with in-person care, although studies in the United States, Canada, and Germany reported the opposite, that is, an increase in medication errors and technological issues such as interrupted sessions [22-25].

Evidence remains sparse; however, on the effects of telemedicine on health care expenditures and health outcomes at the population level. While telemedicine may reduce the cost of particular individual encounters, if it leads to more overall usage, particularly for patients with mild symptoms, it may reduce costs for some subgroups of individuals but still increase total societal costs. A recent US study in the Medicare population found that expanded telemedicine use during the COVID-19 pandemic increased health care usage by 2.7% and health care spending by 1.6% when comparing the highest tertile to the lowest tertile of telemedicine use [26]. In contrast, research on the British National Health Service showed less acute hospital spending on patients registered with a digital-first model than on patients registered at all other practices [27].

The objective of this study is to test the hypothesis that broader adoption of telemedicine can reduce health care costs without compromising health outcomes. The COVID-19 pandemic prompted many countries to relax regulations on the use of telemedicine. As a result, telemedicine use increased from 11% in 2019 to 21% of all doctor consultations in 2020 among Organisation for Economic Co-operation and Development countries [1]. In this study, we leveraged a natural experiment where the Japanese government relaxed regulations for telemedicine use at the start of the COVID-19 pandemic, but different prefectures (local government units, somewhat akin to states in the United States) had differential levels of telemedicine adoption at baseline and were therefore likely to have differential levels of uptake of the new relaxed regulations. Japan is an ideal setting for this quasi-natural experiment for several reasons. First, Japan has a universal health coverage system, and almost the entire population across the country receives the same benefit package regardless of insurance [28-30]. Second, Japan had strict regulations on the use of telemedicine before the pandemic, and the new regulation resulted in a rapid increase in uptake as part of exceptional measures adopted during the pandemic [31]. Finally, there are 47 prefectures in Japan, which we could leverage for the variation in telemedicine use before the relaxation of regulations [32].

## Methods

### Study Design

We used a difference-in-differences (DiD) quasi-experimental design to examine the effects of increased telemedicine use on health expenditures. On April 10, 2020, the Japanese government relaxed restrictions on telemedicine use as an exceptional measure to enable health care providers to reach patients during the COVID-19 pandemic [33]. Before the new regulation, the allowable use of telemedicine in Japan was restricted to patients with chronic diseases and not for first-visit patients, except for emergencies or underserved areas [31]. As a result, telemedicine accounted for only 0.18% of all outpatient medical claims in fiscal year (FY) 2019. After April 2020, telemedicine was permitted for almost all patients, except for those receiving medications that required in-person supervision (eg, immunosuppressant therapy) [33]. Our DiD analysis took advantage of the rapid increase in telemedicine use in FY 2020‐2022 following the relaxation of restrictions in April 2020. We set FY 2017‐2019 as the preintervention period and FY 2020‐2022 as the postintervention period for health expenditure, and FY 2020‐2021 for health outcomes (2022 was the latest year with available data on prefecture telemedicine rates, and 2021 was the latest year with available data on prefecture health outcomes [34]).

Using a median split based on the prefectural population size, the treatment group was defined by the prefecture-wide level of telemedicine usage before the pandemic. Based on the share of telemedicine among outpatient medical claims in FY2019, we defined the top 15 prefectures (representing a population of 62 million, 49.2% of the total population in FY2019) as the treatment group and the remaining 32 prefectures (population of 64 million, 50.8% of the total population) as the control group (Multimedia Appendix 1). A higher-than-median prepandemic share of telemedicine among outpatient medical claims (>0.1624%) was used to define the treatment group, because we expected that health care providers in these prefectures were better prepared to take advantage of the policy change to offer telemedicine services to patients.

### Data and Outcomes

We used publicly available, deidentified data from the total Japanese population to increase sample size and avoid spillover effects. We obtained monthly data at the prefectural level, including health expenditures (inpatient and outpatient), health outcomes (all-cause mortality, glycated hemoglobin [HbA_1c_], systolic blood pressure, diastolic blood pressure, triglycerides, and low-density lipoprotein [LDL] cholesterol) demographics (population size, age, and sex composition), average monthly salary, and annual COVID-19 case rates from government statistics (Multimedia Appendix 2) [35-38]. We obtained the share of telemedicine among total claims by using the medical claim aggregated data published by the Japanese government [34] (Refer to Multimedia Appendix 3 for our detailed calculations).

Our primary outcomes were health expenditures at the total, inpatient, and outpatient levels from FY2017 to FY2022 and health outcomes from 2017 to 2021 at the prefecture level. We focused on prefecture-level statistical data, not individual data, to use a wider range of data—the data of the whole country— and to increase generalizability. Health expenditure data were aggregated from medical claims data published by the Ministry of Health, Labour and Welfare in Japan.

The other outcomes were health outcomes related to common chronic conditions. We focused on health outcomes of all-cause mortality, HbA_1c_, systolic blood pressure, and LDL cholesterol, as these indicators could reflect short-term clinical quality of care [39,40]. For mortality, we used national statistics data. Regarding health outcome data on HbA_1c_, systolic blood pressure, and LDL cholesterol, we used aggregated data from the annual “specific medical checkups” for lifestyle-related diseases conducted for individuals aged 40 to 74. In Japan, all insurers are legally obliged to provide these annual specific checkups to individuals aged 40 to 74 years and report the results to the Ministry of Health, Labour and Welfare [28,41]. These data reflect nationwide health outcomes each year. The aggregated data cover all participants of the checkups, with a large sample size of 29 to 30 million people in total, and the participation rates were stable, ranging from 53.1% to 56.5% each year from 2017 to 2021 [34] (Multimedia Appendix 4). Also, each individual can only receive this specific checkup once a year, ensuring that the data does not include multiple entries for the same individual.

### Statistical Analysis

Data analysis was conducted between October 2023 and December 2024. We used a DiD quasi-natural experiment design. We used 2×2 DiD and DiD within an event-study framework, where we compared pre- versus post-intervention effects year by year from FY2017 to FY2022.

Our main model was the 2×2 (treatment vs control, pre- vs postintervention) DiD approach with 2-way fixed effects to estimate the average effect of the increase in the share of telemedicine on health expenditures and health outcomes. For health expenditures, we used the logarithm of total, inpatient, and outpatient health expenditures per capita in each prefecture as the outcome of this model to mitigate skewness, which means our results indicate the relative percent change in each outcome. Regarding health outcomes, we used the average values of all-cause mortality, HbA_1c_, systolic blood pressure, diastolic blood pressure, and LDL cholesterol in each prefecture. This model used a binary variable for the top 15 prefectures according to their share of telemedicine in FY2019, interacted with as the postintervention period (FY 2020‐2021), which captured the effects of greater telemedicine use on health expenditures.

Models adjusted for population size, share of people aged 65 or older, average monthly income, and new COVID-19 cases per 100,000 population each year. The regression used robust SEs clustered at the prefecture level, and all analyses were weighted according to population size to yield nationally representative estimates for the policy’s impact. Refer to Multimedia Appendix 5 for full regression equations.

Regarding health expenditures, our primary outcome, we also used the DiD approach within an event-study framework to estimate the effects of the intervention over time and to inspect the parallel trend assumption using the following equation. This model used a binary variable for the top 15 prefectures according to their share of telemedicine in FY2019, interacted with each of the 3 postintervention years (from FY2020 to FY2022). Modeling these years separately enabled us to identify the postexpansion trend by year for each outcome relative to the corresponding difference in the reference year (FY2019).

For sensitivity analyses, we used 2 other models. First, we compared prefectures in the top tertile of prepandemic telemedicine use with the bottom tertile of prefectures, rather than splitting the sample based on the median. The top tertile consisted of 8 prefectures with 35.4% of the total population, while the control group included the bottom 27 prefectures with 37.0% of the total population (Multimedia Appendix 6). Second, we examined telemedicine share in FY019 as a continuous treatment variable (ie, without forming treatment or control groups), using an interaction term between the share of telemedicine of each prefecture and a binary variable for the postintervention period (from FY2020 to FY2022). The parameter of interest captures the effects of changes in the share of telemedicine between prefectures in FY2019 on health expenditures following the policy change.

To address the possibility that the parallel trend testing was underpowered compared to our main model, we also adopted a falsification test, which uses the same regression model as our DiD analysis but applies it only to the prepandemic period, comparing 2019 with 2017‐2018.

### Ethical Considerations

The data were publicly accessible, deidentified survey data and were deemed not “human subjects research” by the Harvard T.H. Chan School of Public Health Institutional Review Board (IRB24-0701). The study follows the STROBE (Strengthening the Reporting of Observational Studies in Epidemiology) guidelines.

## Results

### Sample Characteristics

Table 1 presents demographic characteristics of prefectures at baseline, the share of telemedicine (as a percentage of outpatient claims), and COVID-19 cases per 100,000 population each year. The demographic characteristics of the treatment and control prefectures were similar, while the health expenditures per capita, health outcomes, and monthly salary showed only slight differences. COVID-19 cases per 100,000 population were 1.9 times higher in the treatment prefectures compared to the control prefectures in FY2019, when COVID-19 cases were still limited in Japan, but showed little difference in FY2022, when COVID-19 cases increased.

**Table 1.** Characteristics of the study sample, share of telemedicine, and COVID-19 cases.

Characteristic^a^	Treatment prefectures (FY^b^2019 telemedicine share >0.1624%)	Control prefectures (FY2019 telemedicine share <0.1624%)
Number of prefectures	15	32
Population, n	62,158,396	64,280,384
Age group (years), %		
0‐14	11.9	12.4
15‐64	60.7	58.7
≥65	27.4	28.8
Male sex, %	48.4	48.9
Monthly salary (JPY)^c^	349,597	314,668
COVID-19 cases per 100,000^d^		
2019	2.3	1.2
2020	491	258
2021	5886	3776
2022	21,727	21,291
Telemedicine, %^e^		
2017	0.25	0.12
2018	0.24	0.11
2019	0.25	0.12
2020	0.87	0.51
2021	1.08	0.60
2022	1.37	0.89
Health expenditure (JPY)^c^		
Total	254,737	241,739
Inpatient	135,412	126,605
Outpatient	119,327	115,132
Health outcome		
All-cause mortality (per 100,000 population)	1050	1137
BMI (kg/㎡)	23.1	23.3
HbA_1c_^f^ , %	5.6	5.7
Systolic blood pressure (mm Hg)	123	125
Diastolic blood pressure (mm Hg)	75.5	76.0
LDL^g^ cholesterol (mg/dL)	125	124

a Data for the characteristics of the study sample at baseline were collected from Japanese government statistics. Values represent averages from FY2017 to FY2019.

b FY: fiscal year.

c JPY: Japanese yen (US $1=JPY ¥147.67).

d Data on new COVID-19 cases were obtained from the website of the Ministry of Health, Labour and Welfare in Japan.

e The share of telemedicine was calculated among total outpatient medical claims using basic medical fees from the aggregated medical claim data published by the Ministry of Health, Labour and Welfare in Japan.

f HbA_1c_: glycated hemoglobin.

g LDL: low-density lipoprotein.

### Changes in Telemedicine Use

Table 1 shows that after the April 2020 change in regulation, the increase in telemedicine use was much higher in the treatment prefectures compared to control prefectures, being 1.7 times larger in FY2020 (0.87% vs 0.51%), 1.8 times higher in FY2021 (1.08% vs 0.60%), and 1.5 times higher in FY2022 (1.37% vs 0.89%). The increase in the share of telemedicine from FY2019 was larger in the treatment prefectures than in the control prefectures by 0.23 percentage point in FY2020 (0.62% vs 0.39%), 0.35 percentage point in FY2021 (0.83% vs 0.48%), and 0.48 percentage point in FY2021 (1.37% vs 0.89%), reflecting a roughly fourfold increase in telemedicine use by FY2022 compared to the baseline median of 0.16% (IQR 0.13%-0.26%FY2019).

### Results of the Main Model for Changes in Health Expenditure

**Figure 1.**
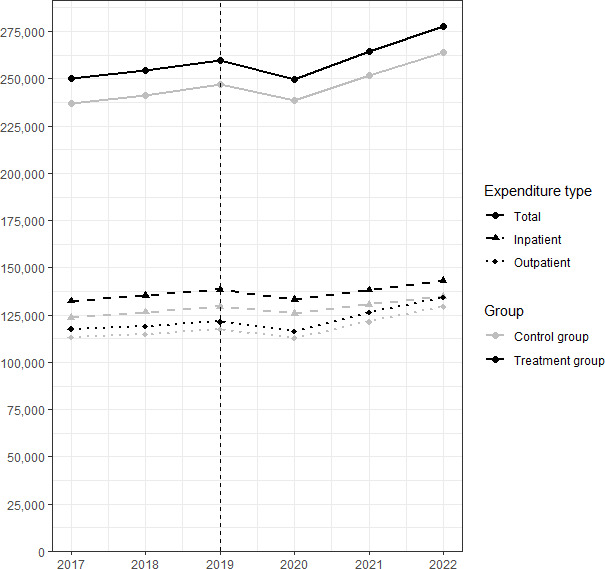
Health expenditure at the total, inpatient, and outpatient levels before and after the intervention. The data were collected from the Japanese government statistics. Each expenditure is the sum of each prefecture’s health expenditure in Japanese yen (US $1=JPY ¥147.67).

Figure 1 shows health expenditure for total, inpatient, and outpatient levels from 2017 to 2022, before and after the intervention.

Table 2 presents 2×2 DiD estimates of changes in health expenditure and health outcomes associated with the increase in the share of telemedicine. The difference in the increased share of telemedicine between the treatment and control prefectures, which corresponds to 0.23 percentage point in FY2020, 0.35 percentage point in FY2021, and 0.48 percentage point in FY2022, was associated with a 1.0% relative decrease (95% CI 0.3%-1.8%) in total health expenditure (*P*=.006) and a 1.1% decrease (95% CI 0.2%-1.9%) in inpatient expenditure (*P*=.02). In absolute terms, a 1% decrease in baseline total health expenditure in the treatment prefectures corresponded to a decrease of 2657 Japanese yen (approximately US $18; 95% CI 789-4525) per patient. Outpatient expenditures did not show a significant difference, −0.8% (95% CI −1.9 to 0.2%; *P*=.11).

**Table 2.** Changes in health expenditure after telemedicine increase based on 2×2 difference-in-differences analyses.

Changes in health expenditure	Baseline in treatment prefectures^a^	Difference-in-differences estimates^b^ (95% CI)^c^	*P* value
Health expenditures (JPY)^d^			
Main model			
Total expenditure	254,737	−0.01 (−0.02 to −0.003)	.006
Inpatient expenditure	135,412	−0.01 (−0.02 to −0.002)	.02
Outpatient expenditure	119,327	−0.01 (−0.02 to 0.002)	.11
Sensitivity 1			
Total expenditure	246,163	−0.01 (−0.02 to 0.000)	.06
Inpatient expenditure	128,388	−0.01 (−0.02 to 0.001)	.06
Outpatient expenditure	117,776	−0.01 (−0.02 to 0.01)	.51
Sensitivity 2			
Total expenditure	—	−0.09 (−0.15 to −0.02)	.008
Inpatient expenditure	—	−0.08 (−0.16 to −0.004)	.04
Outpatient expenditure	—	−0.07 (−0.14 to −0.004)	.04
Health outcome			
All-cause mortality (per 100,000 population)	1050.0	2.6 (−13.5 to 18.6)	.75
HbA_1c_^e^ (%)	5.6	0.0 (0.0 to 0.0)	.47
Systolic blood pressure (mm Hg)	123.0	0.0 (−0.2 to 0.1)	.84
Diastolic blood pressure (mm Hg)	75.5	0.0 (−0.1 to 0.1)	.92
LDL^f^ cholesterol (mg/dL)	125.0	−0.1 (−0.5 to 0.2)	.41

a The numbers of baseline are the average from fiscal year 2017 to fiscal year 2019.

b The difference-in-differences estimates for health expenditures reflect the results of the logarithm of health expenditures.

c CIs are based on the SEs that adjust for clustering at the prefecture level.

d JPY: Japanese yen (US $1=JPY ¥147.67).

e HbA_1c_: glycated hemoglobin.

f LDL: low-density lipoprotein.

**Figure 2.**
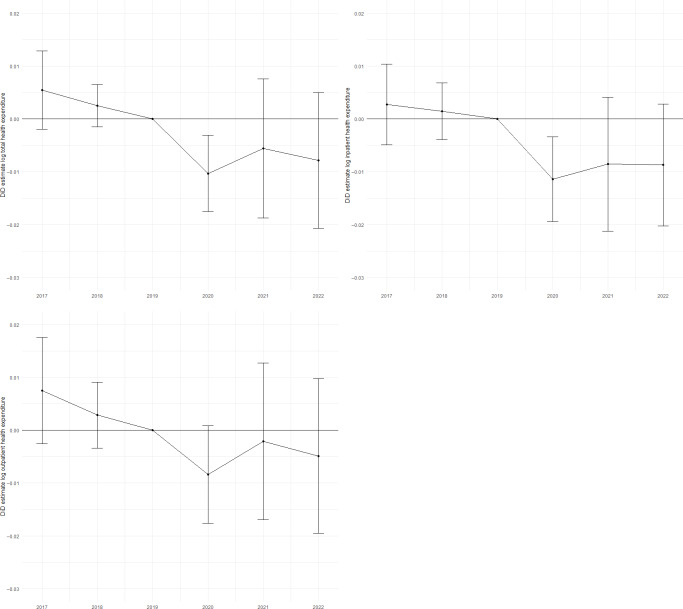
Difference-in-differences (DiD) estimates within the event-study framework.

Figure 2 and Table 3 present the event-study results. There were no significant differences in total, inpatient, and outpatient expenditure in FY2017 and FY2018 compared to FY2019, supporting the parallel trend assumption. In FY2020, expenditure diverged for the treatment and control prefectures; the increased share of telemedicine was associated with a 1.0% (95% CI 0.3%-1.8%; *P*=.006) and a 1.2% (95% CI 0.3%-1.9%; *P*=.005) decrease in total and inpatient health expenditure compared to the reference year (FY2019), respectively. The other outcomes—total and inpatient expenditure in FY2021 and 2022, and outpatient expenditure from FY2020 to FY2022—showed no significant differences.

**Table 3.** Changes in health expenditure based on difference-in-differences within the event-study framework.

Year	Total logarithm of health expenditures (95% CI^a^)	*P* value	Inpatient logarithm of health expenditures (95% CI)	*P* value	Outpatient logarithm of health expenditures (95% CI)	*P* value
2017	0.0055 (−0.002 to 0.013)	.15	0.003 (−0.005 to 0.01)	.48	0.008 (−0.003 to 0.018)	.14
2018	0.003 (−0.002 to 0.007)	.22	0.001 (−0.004 to 0.007)	.59	0.0029 (−0.003 to 0.009)	.37
2019	Reference	Reference	Reference	Reference	Reference	Reference
2020	−0.01 (−0.018 to -0.003)	.006	−0.011 (−0.019 to -0.003)	.005	−0.008 (−0.018 to 0.001)	.08
2021	−0.006 (−0.019 to 0.0075)	.40	−0.009 (−0.021 to 0.004)	.19	−0.002 (−0.017 to 0.0127)	.78
2022	−0.008 (−0.021 to 0.005)	.23	−0.009 (−0.02 to 0.003)	.14	−0.005 (−0.019 to 0.0097)	.51

a CIs are based on the SEs that adjust for clustering at the prefecture level.

### Results of Sensitivity Analyses and Falsification Test for Changes in Health Expenditure

Sensitivity analysis comparing the top tertile to the bottom tertile of prefectures in terms of prepandemic telemedicine use showed similar results: −1.0% (95% CI −1.9% to 0.03%; *P*=.06), ‐1.2% (95% CI −2.4% to 0.1%; *P*=.06), and -0.5% (95% CI −2.0% to 1.0%; *P*=.51) changes in total, inpatient, and outpatient expenditure, respectively.

A sensitivity analysis using the change in telemedicine use as a continuous variable also showed similar patterns. Each 1-percentage point increase in the share of telemedicine in FY2019 was associated with 8.7% (95% CI 2.2% to 16.4%; *P*=.008) and 8.4% (95% CI 0.4% to 16.4%; *P*=.04) decreases in total and inpatient expenditure, respectively. Outpatient expenditures also showed a significant decrease of 7.0% (95% CI 0.4% to 13.7%; *P*=.04), unlike in our main model, where results were nonsignificant (*P*=.11).

The falsification test showed no statistically significant results in total, inpatient, and outpatient health expenditures in FY2019 compared to FY2017 and FY2018 (Multimedia Appendix 7), using the same regression approach as our main model. This finding supports the interpretation that the results of our main model reflect the impact of broader adoption of telemedicine, not preexisting differential trends in health expenditure or regression to the mean.

### Results of Changes in Health Outcomes

Table 2 also presents 2×2 DiD estimates of changes in health outcomes associated with the increase in the share of telemedicine. All health outcomes related to common chronic conditions showed no significant difference.

## Discussion

### Principal Findings

Our analysis of national health expenditure data in Japan during the COVID-19 pandemic showed that prefectures with higher telemedicine use at baseline saw a much larger uptake of telehealth during the pandemic than other prefectures, and this greater telemedicine use was correlated with a decrease in total and inpatient health care expenditures. Notably, these changes occurred despite the relatively small share of visits that were conducted via telehealth even during the pandemic, with differences in telemedicine increases between the treatment and control groups of only 0.23 percentage points in FY 2019‐2020, 0.35 percentage points in FY 2019‐2021, and FY 2019‐2022 (compared to a baseline of 0.16% in FY2019).

The estimates of our sensitivity analysis using the change in telemedicine use as a continuous variable were 8 to 10 times larger than our main model estimates because this model captured the effects of each 1-percentage point increase in the share of telemedicine in FY2019. Considering the gap between the treatment and control groups was 0.13% in FY2019, the results of this model were consistent with the main model’s findings. Overall, these results show the possibility of a larger reduction in health expenditures with the increase in telemedicine use.

Our analysis of health outcomes indicated few adverse effects of telemedicine expansion on health outcomes. Most health outcomes, such as all-cause mortality and HbA_1c_, showed no significant changes due to the increase in telemedicine use. These results suggest that telemedicine expansion in Japan decreased health expenditure without negative impacts on health outcomes.

There are several possible explanations for how increased use of telemedicine could reduce health care expenditures without adverse effects on health outcomes. Telemedicine can enable patients to access care more easily and may prevent excessive usage of more costly care, especially inpatient care. The results of our study are aligned with previous research that showed cost reductions for patients and providers resulting from the use of telemedicine services, and positive safety outcomes compared with in-person care. The results, however, differ from a recent study of Medicare patients that showed increased usage and health spending [26]. A possible explanation is that our research sample included the whole population, including younger people, and substantial differences in baseline levels of telemedicine use (9.5% in the US study vs 0.60% in our study), populations, and health care systems.

The Japanese government ended the exceptional measures adopted during the COVID-19 pandemic at the end of July 2023 and returned to restricting telemedicine use, only allowing family doctors to offer telemedicine for repeat-visit patients or patients living in underserved areas [31,41,42]. Some have raised concerns about patient safety with telemedicine; for example, experts on the Medical Care Subcommittee of the Social Security Council have noted that certain physicians in the cosmetic field might prescribe medications for androgenetic alopecia or obesity through telemedicine, often for commercial purposes, without accompanying protections to address potential medical emergencies [43,44]. Our study was not able to assess quality or access.

### Limitations

Our study has several limitations. First, there may be unobserved confounders influencing both telemedicine adoption and health expenditure and health outcomes, although we used time and prefecture fixed effects to attempt to address this issue and adjusted for factors such as income and COVID-19 incidence. To the best of our knowledge, we are unaware of any other large-scale policies that took effect differentially between the treatment and control groups during this period, other than telemedicine adoption. Most health policies during the COVID-19 pandemic were implemented nationwide, a state of emergency was declared in urban areas, and revisions of medical reimbursement fees and school closures were implemented in all prefectures, which influenced both the treatment and control groups. Nonetheless, there are some possibilities that prefectures in the treatment group adopted different health policies than prefectures in the control group during the pandemic period. Second, we lack data on the quality of care or patient satisfaction and were only able to analyze usage, spending, and annual medical checkups for people aged 40 to 74 years, whose participation rates were around 55%. We also could not assess potential disparities in access to telemedicine across populations, given the prefecture-level nature of our data. Furthermore, our health outcomes are limited to the available statistical data, such as all-cause mortality and medical checkup results, meaning that we were unable to assess other health outcomes. Finally, our results may not generalize to nations with higher telemedicine usage rates than Japan.

### Conclusions

In this nationwide study of Japan during the COVID-19 pandemic, we found a significant decrease in health care spending associated with the increased adoption of telemedicine, with little adverse influence on health outcomes. Although further research is needed on the safety of telemedicine, for policymakers considering whether to expand or restrict telemedicine, our study suggests that telemedicine usage may produce benefits for cost containment in some contexts.

## Supplementary material

10.2196/72051 Multimedia Appendix 1 Prefectures in treatment and control groups, share of telemedicine from FY2017 to FY2022, and population in FY2019. FY: fiscal year.

10.2196/72051 Multimedia Appendix 2 Data resources.

10.2196/72051 Multimedia Appendix 3 Calculation of the share of telemedicine in each prefecture.

10.2196/72051 Multimedia Appendix 4 The participation rate in specific health checkups.

10.2196/72051 Multimedia Appendix 5 Regression equations.

10.2196/72051 Multimedia Appendix 6 Baseline data of sensitivity analysis.

10.2196/72051 Multimedia Appendix 7 Regression results of falsification test.

10.2196/72051 Checklist 1 STROBE checklist.

## Abbreviations

DiD: difference-in-differences
FY: fiscal year
HbA_1c_: glycated hemoglobin
LDL: low-density lipoprotein
STROBE: Strengthening the Reporting of Observational Studies in Epidemiology
