# Supplementary material for: Impact of Telemedicine on Health Expenditures During the COVID-19 Pandemic in Japan: Quasi-Experimental Study
Source: J Med Internet Res. 2025 Sep 23;27:e72051. doi: 10.2196/72051 (PMC12456874; doi:10.2196/72051)
Supplement: Multimedia Appendix 1 [file jmir-v27-e72051-s001.docx]

# Multimedia Appendix 1. Prefectures in Treatment and Control Groups, Share of telemedicine form FY 2017 to 2022, and Population in FY 2019

| Rank of Telemedicine in FY 2019 | Prefecture | Share of Telemedicine (%) | | | | |  | Population in FY 2019 | Main Model | Sensitivity Analysis |
| --- | --- | --- | --- | --- | --- | --- | --- | --- | --- | --- |
|  |  | FY 2017 | FY 2018 | FY 2019 | FY 2020 | FY 2021 | FY  2022 |  |  |  |
| 1 | Tokyo | 0.298 | 0.286 | 0.305 | 1.381 | 1.857 | 2.265 | 13,920,663 | Treatment | Treatment |
| 2 | Tokushima | 0.312 | 0.295 | 0.295 | 0.605 | 0.835 | 1.743 | 727,977 | Treatment | Treatment |
| 3 | Kyoto | 0.289 | 0.281 | 0.292 | 0.835 | 1.065 | 1.417 | 2,582,957 | Treatment | Treatment |
| 4 | Kanagawa | 0.267 | 0.253 | 0.269 | 0.973 | 1.256 | 1.460 | 9,198,268 | Treatment | Treatment |
| 5 | Hiroshima | 0.260 | 0.256 | 0.258 | 0.634 | 0.736 | 1.026 | 2,804,177 | Treatment | Treatment |
| 6 | Osaka | 0.255 | 0.251 | 0.250 | 0.670 | 0.871 | 1.066 | 8,809,363 | Treatment | Treatment |
| 7 | Ehime | 0.258 | 0.249 | 0.245 | 0.614 | 0.624 | 0.715 | 1,339,215 | Treatment | Treatment |
| 8 | Hokkaido | 0.218 | 0.195 | 0.240 | 0.917 | 0.936 | 1.069 | 5,250,049 | Treatment | Treatment |
| 9 | Fukuoka | 0.251 | 0.231 | 0.230 | 0.797 | 0.786 | 1.018 | 5,103,679 | Treatment | - |
| 10 | Wakayama | 0.241 | 0.224 | 0.225 | 0.386 | 0.507 | 0.928 | 924,933 | Treatment | - |
| 11 | Hyogo | 0.217 | 0.195 | 0.192 | 0.598 | 0.709 | 0.887 | 5,466,190 | Treatment | - |
| 12 | Kochi | 0.147 | 0.149 | 0.181 | 0.740 | 0.566 | 0.694 | 698,029 | Treatment | - |
| 13 | Nagano | 0.157 | 0.167 | 0.171 | 0.632 | 0.559 | 0.761 | 2,048,790 | Treatment | - |
| 14 | Okayama | 0.200 | 0.175 | 0.169 | 0.533 | 0.679 | 1.475 | 1,889,586 | Treatment | - |
| 15 | Yamaguchi | 0.170 | 0.165 | 0.162 | 0.358 | 0.367 | 0.538 | 1,358,336 | Treatment | - |
| 16 | Chiba | 0.158 | 0.148 | 0.162 | 0.735 | 0.853 | 0.944 | 6,259,382 | Control | - |
| 17 | Ishikawa | 0.185 | 0.157 | 0.160 | 1.144 | 1.003 | 1.160 | 1,137,649 | Control | - |
| 18 | Saitama | 0.157 | 0.150 | 0.160 | 0.708 | 0.899 | 1.098 | 7,349,693 | Control | - |
| 19 | Gifu | 0.166 | 0.149 | 0.156 | 0.525 | 0.480 | 0.783 | 1,986,587 | Control | - |
| 20 | Shimane | 0.130 | 0.128 | 0.145 | 0.497 | 0.501 | 1.186 | 674,346 | Control | - |
| 21 | Aichi | 0.150 | 0.140 | 0.144 | 0.514 | 0.716 | 1.006 | 7,552,239 | Control | Control |
| 22 | Gunma | 0.219 | 0.173 | 0.144 | 0.515 | 0.530 | 0.753 | 1,942,456 | Control | Control |
| 23 | Yamanashi | 0.168 | 0.146 | 0.143 | 0.543 | 0.634 | 0.984 | 810,956 | Control | Control |
| 24 | Kagawa | 0.144 | 0.139 | 0.143 | 0.409 | 0.390 | 0.508 | 956,347 | Control | Control |
| 25 | Oita | 0.131 | 0.126 | 0.139 | 0.524 | 0.477 | 0.631 | 1,135,434 | Control | Control |
| 26 | Mie | 0.150 | 0.138 | 0.129 | 0.421 | 0.553 | 1.009 | 1,780,882 | Control | Control |
| 27 | Nara | 0.133 | 0.119 | 0.127 | 0.450 | 0.476 | 0.591 | 1,330,123 | Control | Control |
| 28 | Kagoshima | 0.153 | 0.141 | 0.126 | 0.451 | 0.480 | 0.775 | 1,602,273 | Control | Control |
| 29 | Shiga | 0.109 | 0.089 | 0.103 | 0.517 | 0.575 | 0.857 | 1,413,943 | Control | Control |
| 30 | Shizuoka | 0.113 | 0.098 | 0.101 | 0.291 | 0.511 | 1.121 | 3,643,528 | Control | Control |
| 31 | Nagasaki | 0.111 | 0.103 | 0.101 | 0.459 | 0.449 | 0.645 | 1,326,524 | Control | Control |
| 32 | Kumamoto | 0.103 | 0.094 | 0.100 | 0.450 | 0.524 | 1.072 | 1,747,567 | Control | Control |
| 33 | Tochigi | 0.097 | 0.097 | 0.099 | 0.456 | 0.514 | 0.677 | 1,933,990 | Control | Control |
| 34 | Fukui | 0.062 | 0.049 | 0.098 | 0.722 | 0.385 | 0.779 | 767,937 | Control | Control |
| 35 | Saga | 0.094 | 0.094 | 0.092 | 0.345 | 0.347 | 0.494 | 814,711 | Control | Control |
| 36 | Tottori | 0.092 | 0.090 | 0.083 | 0.419 | 0.420 | 0.841 | 555,558 | Control | Control |
| 37 | Miyagi | 0.095 | 0.083 | 0.082 | 0.414 | 0.412 | 0.635 | 2,306,365 | Control | Control |
| 38 | Miyazaki | 0.075 | 0.065 | 0.080 | 0.345 | 0.403 | 0.707 | 1,073,301 | Control | Control |
| 39 | Ibaraki | 0.083 | 0.074 | 0.078 | 0.482 | 0.503 | 0.730 | 2,860,307 | Control | Control |
| 40 | Okinawa | 0.076 | 0.072 | 0.076 | 0.515 | 0.769 | 0.828 | 1,453,168 | Control | Control |
| 41 | Fukushima | 0.088 | 0.073 | 0.071 | 0.472 | 0.440 | 0.734 | 1,845,519 | Control | Control |
| 42 | Toyama | 0.079 | 0.069 | 0.070 | 0.644 | 0.448 | 0.702 | 1,043,502 | Control | Control |
| 43 | Niigata | 0.066 | 0.065 | 0.067 | 0.351 | 0.356 | 0.707 | 2,223,106 | Control | Control |
| 44 | Yamagata | 0.072 | 0.066 | 0.065 | 0.404 | 0.678 | 1.446 | 1,077,666 | Control | Control |
| 45 | Akita | 0.059 | 0.058 | 0.057 | 0.233 | 0.410 | 1.209 | 966,490 | Control | Control |
| 46 | Iwate | 0.044 | 0.042 | 0.047 | 0.374 | 0.463 | 0.620 | 1,226,816 | Control | Control |
| 47 | Aomori | 0.019 | 0.019 | 0.022 | 0.308 | 0.462 | 0.665 | 1,246,371 | Control | Control |

For our main model, we employed a median split based on the prefectural population size to form the treatment and control prefectures based on the share of telemedicine among outpatient medical claims in FY 2019. (See Table S1 for specific prefectures included in the treatment and control group.)

The top 15 prefectures in terms of telemedicine use prior to the COVID-19 pandemic (49.2% of the total population) were assigned to the treatment group, while the bottom 32 prefectures in terms of telemedicine use prior to 2020 were assigned to the control group (50.8%).

For sensitivity analyses, we used a tertile split based on the prefectural population size to form the treatment and control prefectures based on the share of telemedicine among outpatient medical claims in FY 2019. The treatment group was defined as the top tertile of prefectures with respect to telemedicine use prior to the pandemic, comprising 8 prefectures (35.38% of the total population). The control group was defined as the bottom tertile of prefectures with respect to telemedicine us prior to the pandemic, comprising 27 prefectures (36.96% of the total population).
