# Supplementary material for: Impact of Telemedicine on Health Expenditures During the COVID-19 Pandemic in Japan: Quasi-Experimental Study
Source: J Med Internet Res. 2025 Sep 23;27:e72051. doi: 10.2196/72051 (PMC12456874; doi:10.2196/72051)
Supplement: Multimedia Appendix 2 [file jmir-v27-e72051-s002.docx]

# Multimedia Appendix 2. Data Resources

We used data from the total Japanese population to maximize the sample size and avoid spillover effects. We obtained monthly data at the prefectural level from the following government statistics.

- Data on health expenditures (inpatient and outpatient) from “Estimates of National Medical Care Expenditure” on <https://www.e-stat.go.jp/stat-search/files?page=1&toukei=00450032&tstat=000001020931>
- Data on demographics (population size, age and sex composition) from “Population Estimates” on <https://www.e-stat.go.jp/en/stat-search/files?page=1&toukei=00200524&tstat=000000090001>
- Data on average monthly salary from “Basic Survey on Wage Structure” on <https://www.e-stat.go.jp/stat-search/files?page=1&toukei=00450091&tstat=000001011429>
- Data on COVID-19 new cases per day from the website of the Ministry of Health, Labour and Welfare on <https://covid19.mhlw.go.jp/>
- Data on the medical claim aggregated data and health outcomes aggregated data from “National Database of Health Insurance Claims and Specific Health Checkups of Japan Open Data” on the website of the Ministry of Health, Labour and Welfare on <https://www.mhlw.go.jp/stf/seisakunitsuite/bunya/0000177182.html>
- Data on mortality from “Vital Statistics” on <https://www.e-stat.go.jp/en/stat-search/files?page=1&toukei=00450011&tstat=000001028897>

All of this data is publicly available through open access, and anyone can access it.
