# Supplementary material for: Impact of Telemedicine on Health Expenditures During the COVID-19 Pandemic in Japan: Quasi-Experimental Study
Source: J Med Internet Res. 2025 Sep 23;27:e72051. doi: 10.2196/72051 (PMC12456874; doi:10.2196/72051)
Supplement: Multimedia Appendix 3 [file jmir-v27-e72051-s003.docx]

# Multimedia Appendix 3. Calculation of Share of Telemedicine in Each Prefecture

We calculated the share of telemedicine usage in each prefecture, using aggregated national medical claims data from FY 2017 to 2022 on the website of the Ministry of Health, Labour and Welfare in Japan. The medical claims data include the number of occasions different type of treatment was provided by prefecture each year.

For the denominator, we used the number of claims recorded in the basic medical claims section (“Kihon Sinryo Ryo”). Patients pay either a fee for their first visit (“Shoshin Ryo”), a second-visit fee for hospitals with less than 200 hospital beds (“Saishin Ryo”), second-visit fee for hospitals with more than 200 hospital beds (“Gairai Shinryo Ryo”), or a telemedicine visit fee ("Online Shinryo Ryo”), which accounted for 15%, 73%, 12%, and 0.001% of all claims in FY 2021, respectively.

The telemedicine visit fee (Online Shinryo Ryo) is only charged during consultations for patients with chronic diseases. Patients who made a video consultation or telephone call when their symptoms got worse paid the second-visit fee for a video conversation or telephone call (“Denwato Saisin Ryo”), instead of the telemedicine visit fee (Online Shinryo Ryo). We counted the total number of telemedicine consultations (our numerator) by summing the number of first-visits for telemedicine plus second-visits for a video conversation or telephone call.
