# Supplementary material for: Impact of Telemedicine on Health Expenditures During the COVID-19 Pandemic in Japan: Quasi-Experimental Study
Source: J Med Internet Res. 2025 Sep 23;27:e72051. doi: 10.2196/72051 (PMC12456874; doi:10.2196/72051)
Supplement: Multimedia Appendix 4 [file jmir-v27-e72051-s004.docx]

# Multimedia Appendix 4. The participation rate in specific health checkups

In Japan, all insurers are legally obliged to provide the annual “specific medical check-ups” to the population aged 40 to 74 and report the results to the Ministry of Health, Labour and Welfare.

|  | Number of individuals eligible for checkups | Number of individuals who received checkups | % |
| --- | --- | --- | --- |
| 2017 | 53,876,463 | 28,587,618 | 53.1% |
| 2018 | 53,723,213 | 29,396,195 | 54.7% |
| 2019 | 53,798,756 | 29,935,810 | 55.6% |
| 2020 | 54,183,746 | 28,939,947 | 53.4% |
| 2021 | 53,801,976 | 30,389,789 | 56.5% |
