# Supplementary material for: Impact of Telemedicine on Health Expenditures During the COVID-19 Pandemic in Japan: Quasi-Experimental Study
Source: J Med Internet Res. 2025 Sep 23;27:e72051. doi: 10.2196/72051 (PMC12456874; doi:10.2196/72051)
Supplement: Multimedia Appendix 5 [file jmir-v27-e72051-s005.docx]

# Multimedia Appendix 5. Regression Equations

Our main model, the $2\times2$ (treatment vs. control, pre vs. post intervention) DiD approach with two-way fixed effects to estimate the average effect of the increase in the share of telemedicine on health expenditures using the following equation:

$${Log Total, Outpatient, or Inpatient Health Expenditure/Health outcomes per person}_{it}=\beta{Treat}_{i}{Post}_{t}+\gamma{Prefecture}_{\boldsymbol{i}}\boldsymbol{+}\theta{Year}_{t}+\alpha_{1}Populati{on}_{it}+\alpha_{2}{Elderly Population Share}_{it}+\alpha_{3}{Salary}_{it}+\alpha_{4}{COVID 19 cases (per 100,000)}_{it}+\varepsilon_{it}$$

where $i$ and $t$ respectively index prefecture and year. $Treat$ and $Post$ denote dummy variables; $Treat$ is equal to 1 for the top 15 Prefectures according to their share of telemedicine in FY 2019 and $Post$ is equal to 1 in FY 2020 and 2021. We adjusted for population size, share of elderly people aged 65 or over, monthly salary, and COVID-19 new cases per 100,000 population. The regression used robust standard errors clustered at the prefecture level, and all analyses were weighted according to population size to yield nationally-representative estimates for the policy’s impact.

To address the possibility that the parallel trend testing was underpowered compared to our main model, we also adopted falsification test, which uses the same regression model as our difference-in-difference but applies it only to the pre-pandemic period, comparing 2019 to 2017-2018.

We also used the DiD approach within an event-study framework to estimate the effects of the intervention over time and to inspect the parallel trend assumption using the following equation:

$${Log Total, Outpatient, or Inpatient Health Expenditure per person}_{it}=\beta_{1}{Treat}_{i}{Year}_{2017}+\beta_{2}{Treat}_{i}{Year}_{2018}+\beta_{3}{Treat}_{i}{Year}_{2020}+\beta_{4}{Treat}_{i}{Year}_{2021}+\beta_{5}{Treat}_{i}{Year}_{2022}+\gamma{Prefecture}_{\boldsymbol{i}}\boldsymbol{+}\theta{Year}_{t}+\alpha_{1}Populati{on}_{it}+\alpha_{2}{Elderly Population Share}_{it}+\alpha_{3}{Salary}_{it}+\alpha_{4}{COVID 19 cases (per 100,000)}_{it}+\varepsilon_{it}$$

Our parameters of interest are $\beta_{v}$, which capture the temporal effects of the intervention by comparing the difference in outcomes between intervention and control groups to the corresponding difference in the reference year (FY 2019).

For our second sensitivity analysis, we examined telemedicine share in FY 2019 as a continuous treatment variable (i.e., without forming treatment or control groups) using the following equation:

$${Log Total, Outpatient, or Inpatient Health Expenditure per person}_{it}=\beta{Telemedicine Share in FY 2019}_{i}\times{Post}_{t}+\gamma{Prefecture}_{\boldsymbol{i}}\boldsymbol{+}\theta{Year}_{t}+\alpha_{1}Populati{on}_{it}+\alpha_{2}{Elderly Population Share}_{it}+\alpha_{3}{Salary}_{it}+\alpha_{4}{COVID 19 cases (per 100,000)}_{it}+\varepsilon_{it}$$

The parameter of interest is $\beta$, which captures the effects of changes in the share of telemedicine between prefectures in FY 2019 on health expenditure following the policy change.
