# Supplementary material for: Impact of Telemedicine on Health Expenditures During the COVID-19 Pandemic in Japan: Quasi-Experimental Study
Source: J Med Internet Res. 2025 Sep 23;27:e72051. doi: 10.2196/72051 (PMC12456874; doi:10.2196/72051)
Supplement: Multimedia Appendix 6 [file jmir-v27-e72051-s006.docx]

# Multimedia Appendix 6. Baseline data of Sensitivity Analysis

|  | **Treatment Prefectures** (Upper tertile: share of telemedicine in FY 2019 higher than 0.23%) | **Control Prefectures** (Lower tertile: share of telemedicine in FY 2019 lower than 0.145%) |
| --- | --- | --- |
| Number of prefectures | 8 | 27 |
| Population^a^ | 44,595,668 | 46,877,704 |
| Age group (%)^a^ |  |  |
| 0-14 yr | 11.8% | 12.4% |
| 15-64 yr | 62.4% | 58.2% |
| 65- yr | 25.8% | 29.4% |
| Male sex (%)^a^ | 48.9% | 48.6% |
| Health Expenditure (JPY)^a^ | |  |
| Total | 246,163 | 248,890 |
| Inpatient | 128,388 | 131,393 |
| Outpatient | 117,776 | 117,497 |
| Monthly Salary (JPY)^a^ | 362,947 | 309,572 |
| Telemedicine (%)^b^ |  |  |
| in 2017 | 0.27% | 0.11% |
| in 2018 | 0.26% | 0.10% |
| in 2019 | 0.27% | 0.10% |
| in 2020 | 0.98% | 0.44% |
| in 2021 | 1.25% | 0.53% |
| in 2022 | 1.54% | 0.84% |
| COVID-19 New Cases per 100,000 population^c^ | |  |
| in 2019 | 2.6 | 0.9 |
| in 2020 | 575 | 206 |
| in 2021 | 6,501 | 3,401 |
| in 2022 | 21,394 | 22,028 |

a The data for the characteristics of the study sample at baseline were collected from the Japanese government statistics. The numbers are the average from FY 2017 to FY 2019.

b We calculated the share of telemedicine among total outpatient medical claims using basic medical fees of the medical claim aggregated data published by the Ministry of Health, Labour and Welfare in Japan.

c The data on COVID-19 new cases were collected from the website of the Ministry of Health, Labour and Welfare in Japan.
