# Supplementary material for: Impact of Telemedicine on Health Expenditures During the COVID-19 Pandemic in Japan: Quasi-Experimental Study
Source: J Med Internet Res. 2025 Sep 23;27:e72051. doi: 10.2196/72051 (PMC12456874; doi:10.2196/72051)
Supplement: Multimedia Appendix 7 [file jmir-v27-e72051-s007.docx]

# Multimedia Appendix 8. Regression results of falsification test

The below table presents $2\times2$ DiD estimates of changes in the logarithm of health expenditures with the increase in FY 2019. All estimates showed no significant difference, which means there is no significant changes in health expenditures between treatment and control group in FY 2019.

This result can support that the results of our main model adequately reflect the impact of broader adoption of telemedicine, not the long-term trend of health expenditure or regression to the mean.

| The logarithm of Health Expenditures | Difference-in-differences estimates (95% CI) | *P* value |
| --- | --- | --- |
| Total Expenditure | -0.001 (-0.006 to 0.003) | 0.563 |
| Inpatient Expenditure | -0.001 (-0.008 to 0.006) | 0.808 |
| Outpatient Expenditure | -0.003 (-0.007 to 0.001) | 0.184 |
